# *FMR1* and AKT/mTOR Signaling in Human Granulosa Cells: Functional Interaction and Impact on Ovarian Response

**DOI:** 10.3390/jcm10173892

**Published:** 2021-08-30

**Authors:** Julia Rehnitz, Edison Capp, Birgitta Messmer, Xuan Phuoc Nguyen, Ariane Germeyer, Alexander Freis, Jens Erik Dietrich, Karin Hinderhofer, Thomas Strowitzki, Peter H. Vogt

**Affiliations:** 1 Division of Reproductive Genetics, Department of Gynecological Endocrinology and Fertility Disorders, University Women’s Hospital Heidelberg, 69120 Heidelberg, Germany; birgitta.messmer@med.uni-heidelberg.de (B.M.); XuanPhuoc.Nguyen@med.uni-heidelberg.de (X.P.N.); peter.vogt@med.uni-heidelberg.de (P.H.V.); 2 Department of Gynecological Endocrinology and Fertility Disorders, University Women’s Hospital Heidelberg, 69120 Heidelberg, Germany; 00010315@ufrgs.br (E.C.); Ariane.Germeyer@med.uni-heidelberg.de (A.G.); alexander.freis@med.uni-heidelberg.de (A.F.); jens.dietrich@med.uni-heidelberg.de (J.E.D.); thomas.strowitzki@med.uni-heidelberg.de (T.S.); 3 Department of Obstetrics and Gynecology, Medicine School, Universidade Federal do Rio Grande do Sul, Porto Alegre 90035-003, Brazil; 4 Laboratory of Molecular Genetics, Institute of Human Genetics, University Heidelberg, 69120 Heidelberg, Germany; karin.hinderhofer@med.uni-heidelberg.de

**Keywords:** *FMR1*, granulosa cells, *AKT1*, *FOXO3*, *FOXO1*, *mTOR*, *S6K*, *TSC2*, ovarian response

## Abstract

We aimed to determine whether a functional link with impact on female ovarian reserve exists between *FMR1* expression and expression ratios of AKT/mTOR signaling genes in human granulosa cells in vivo, as suggested from prior in vitro data. Three hundred and nine women, who were classified as normal (NOR; *n* = 225) and poor (POR; *n* = 84) responders based on their ovarian reserve, were recruited during stimulation for assisted reproductive techniques. Expressions of *FMR1* and of key genes of the AKT/mTOR and AKT/FOXO1/3 signaling pathways were comparatively analyzed in their granulosa cells. *FMR1* expression in granulosa cells of NOR and POR correlated significantly with *AKT1, TSC2, mTOR,* and *S6K* expression. No correlation was found between *FMR1* and *FOXO1* in all, and *FOXO3* expression in POR, patients. *AKT1* expression was significantly higher and *FOXO1* expression lower in POR samples, whereas *AKT1* expression was lower and *FOXO1* expression was higher in NOR samples. In human native granulosa cells, *FMR1* expression significantly correlated with the expression of key genes of the AKT/mTOR signaling pathway, but not with the FOXO1/3 signaling pathway. Our data point to a functional link between *FMR1* expression and expression of the AKT/mTOR signaling pathway genes controlling human follicular maturation.

## 1. Introduction

Controlled ovarian follicular maturation (folliculogenesis) after puberty is essential for successful reproduction. It results in an individual ovarian reserve dependent on age [1]. Related disorders are diminished ovarian reserve (DOR) and premature ovarian insufficiency (POI), which significantly influence spontaneous pregnancy rates and success rates in assisted reproductive techniques (ART) [2]. When women with DOR undergo controlled ovarian stimulation during ART, there is an increased risk of poor ovarian response leading to a reduced number of mature follicles. Normal responders (NOR) and poor responders (POR) are distinguished according to the ESHRE Bologna guidelines [3].

A number of genes and signaling pathways are presumed to play crucial roles in the control of follicular maturation and the ovarian reserve [4]. Of these, *FMR1* (Fragile X-Mental Retardation 1, OMIM: *309550), located on the X chromosome (Xq27.3) [5], is the most prominent, because of its high mutation frequency in the female germline. Its protein FMRP is mainly localized in the granulosa cells (GCs) of ovarian follicles at different stages [6]. The gene contains a CGG base triplet in its 5´UTR (untranslated region) with usually 26–34 repeats in distinct human populations [7]. CGG triplet repeats between 54 and 200 are termed as “premutation” (PM), because this longer CGG block becomes subsequently unstable during further heredity and may lead to transmission of a full mutation (FM) allele with >200 CGG repeats in the next generation. In such cases, *FMR1* is silenced due to complete CpG methylation of its promoter domain, finally causing fragile X syndrome (FXS; OMIM #300624) [8]. Up to 20% of women with *FMR1*-PM and so-called “grey zone” alleles (45–55 repeats) are affected by POI [9,10], making it the most common genetic cause of POI or premature ovarian failure (POF), also called fragile X POI (FXPOI, OMIM #311360) [11]. POI/POF is defined as severe or complete ovarian exhaustion before the age of 40 with hypergonadotropic oligo- to amenorrhea lasting longer than four months [12]. Approximately 1% of women are affected by this, and, in 20–25%, a genetic component is suggested [13]. Women with a *FMR1*-PM allele produce elevated *FMR1*-mRNA levels, causing reduced FMRP levels due to a negative feedback loop mechanism [14]. Increased *FMR1* expression thus appears to be an important ovarian pathomechanism [15,16]. Accordingly, elevated *FMR1* expression analyzed in native GCs of women with distinct ovarian responses demonstrated an association with poor ovarian reserve in women with CGG repeats below the norm of 26–34 repeats [17]. Additionally, variable *FMR1* genotypes with CGG repeats below or above 26–34 impact ovarian reserve [18,19,20]. Recently, we discovered that the *FMR1* transcription rate in human GCs is dependent on epigenetic factors, namely, CpG methylation patterns in three distinct genomic regions, designated as VMR1–3 (variably methylated regions) [21].

The AKT/mTOR (AKT serine/threonine kinase/mechanistic Target of Rapamycin) signaling pathway controls early folliculogenesis by maintaining the primordial follicular pool. This includes primordial follicular activation, GC proliferation, and oocyte–GC and inter-GC communication control [22,23,24]. Moreover, the pathway is active during later gonadotropin-dependent follicular maturation until preovulatory follicle formation. In animal models dependent on FSH ligation to its receptor, follicular activation through activation of this pathway has been demonstrated [25,26]. Three isoforms of AKT that share approximately 80% of their amino acids and are widely expressed in various tissues are known. The role of *Akt3* in the ovary is unclear. *Akt2*-deficient mice show normal fertility and *Akt1*-deficient female mice are reported to have reduced fertility [27].

*AKT1* expression has been identified as a marker in cumulus cells for a positive pregnancy outcome in humans [28]. AKT is involved in mTOR signaling; it induces phosphorylation of tuberous sclerosis protein 2 (TSC2), which forms a dimer with tuberous sclerosis protein 1 (TSC1) and triggers mTOR complex (mTORC) formation. mTORC phosphorylates, and thereby activates, S6K (ribosomal protein S6 kinase), which is the key control element for cellular transcript translation and protein synthesis. Likewise, AKT regulates nuclear transcription factors termed forkhead box O (FoxOs). While three FoxOs (FoxO1, 3, and 4) are broadly expressed, a fourth (FoxO6) is more limited to the brain [29]. In animal studies, FoxO1 and O3 have been shown to be important for gametogenesis in mammals. They are highly expressed in GCs [30] and, in their unphosphorylated form, suppress ovarian follicular activation at the earliest stages of follicular growth, avoiding accelerated follicular initiation. Further GC proliferation is inhibited and a proapoptotic pathway is initiated [31].

Previously, in the COV434 granulosa cell line, we described a putative functional linkage of *FMR1*/FMRP expression with the expression of key genes of the AKT/mTOR signaling pathway. Moreover, *AKT1*, *mTOR*, *S6K*, and *FMR1*/FMRP expression levels were altered depending on rFSH (recombinant follicle-stimulating hormone) stimulation [32].

Further support of the functional linkage between *FMR1* expression and genes of the AKT/mTOR signaling pathway is indicated in mice by the binding of *Tsc2* and *mTor* with FMRP within the RNA-Interference Silence Complex (RISC) [33]. RISCs locate target RNAs, leading to translational repression and/or epigenetic silencing [34]. Moreover, *Fmr1* knockdown causes elevated mTOR signaling [35], and S6K is a major FMRP-phosphorylating enzyme in human neuronal cells [36]. Additionally, in mouse ovary, *Fmr1*-PM was associated with increased AKT phosphorylation [37].

In this study, we aimed to confirm our earlier COV434 cell culture experiments using native GCs from women undergoing ART at our outpatient clinic. Our analysis of *FMR1* expression and of key genes of the AKT/mTOR and AKT/FOXO1/3 signaling pathways in native GCs of 309 women with distinct ovarian responses also indicates a functional linkage between the AKT/mTOR signaling key genes and *FMR1* in vivo.

## 2. Materials and Methods

### 2.1. Design and Patients

We performed a prospective study and enrolled 309 women with different ovarian responses at the Heidelberg University Women’s Hospital (Germany) from February 2013 to October 2019. They all provided written informed consent and completed a clinical questionnaire for collecting blood and GC samples. The study was approved by the local ethics committee of the Ruprecht Karls University of Heidelberg, Germany (number S-602/2013) and conducted according to the principles expressed in the Declaration of Helsinki.

Clinical and demographic information was obtained from medical records and questionnaires (age at presentation; body mass index (BMI); baseline hormone levels: serum follicle-stimulating hormone (FSH), luteinizing hormone (LH), estradiol (E2), and anti-Müllerian hormone (AMH); and reproductive parameters: antral follicle count (AFC), total number of oocytes, and total number of mature (MII) oocytes). According to the ESHRE Bologna Criteria [3], patients were classified as normal responders (NOR) (*n* = 225) or poor responders (POR) (*n* = 84).

### 2.2. Ovarian Stimulation 

The appropriate protocol was selected by physicians independently of this study. Either the long protocol of gonadotropin-releasing hormone (GnRH) agonist administration (long GnRH agonist protocol) or the GnRH antagonist protocol was used for ovarian stimulation. For the long GnRH agonist protocol, an initial downregulation, using a GnRH agonist at day 20 + 1 of the menstrual cycle, was used. On day two of the following cycle, gonadotropins (rFSH or HMG: human menopausal gonadotropin) were injected daily to induce proper follicular maturation. When the follicles attained a diameter of 17 mm, ovulation was induced by human chorionic gonadotropin injection and the oocytes were retrieved after 36 hours, using ultrasound-guided follicular puncture. Aspirates then were placed in 14 mL round-bottom tubes containing phosphate-buffered saline (ART-4012, SAGE IVF, Cooper Surgical, Trumbull, CT, USA) and heparin (2.5 IU/mL) or Flush medium (4 GM 501F-100, Gynemed, Lensahn, Germany). For the GnRH antagonist protocol, gonadotropins (mainly recFSH or HMG) were injected daily to induce proper follicular maturation, beginning at day two of the menstrual cycle. When the leading follicle reached an average diameter of 14 mm, a GnRH antagonist was used to prevent preterm spontaneous ovulation. When the follicles reached 18 mm in diameter, the oocytes were retrieved after induction of ovulation as described above. The cumulative dose of gonadotropins was determined based on the patients’ responses.

### 2.3. Retrieval of Granulosa Cells

GCs were recovered from the follicular fluid after transvaginal ultrasound-guided follicle puncture for in vitro fertilization (IVF), as described previously [17]. The follicles were aspirated with a specific needle (Premium Fas Single Lumen, #4551 NS-AS1; Gynétics Medical Products N.V., Lommel, Belgium) that was connected to a vacuum pump (Cook Medical, K-MAR-5200, Bloomington, IN, USA). The aspirated follicular fluid was collected in 14 mL round-bottom tubes (Falcon, NY 352001, USA) and maintained at 37 °C. The follicular fluid was then transferred to a cell culture dish (150350 or 150360, Thermo Fisher Scientific, Nunc, Waltham, MA, USA) on a table heated to 37 °C (Workstation L126 Dual, K-Systems, Birkerød, Denmark). Mural GCs were identified morphologically within the follicular fluid using a Nikon SMZ1500 zoom-stereomicroscope (Nikon Instruments Europe B.V., Amsterdam, The Netherlands). In most cases, GCs were directly taken from the follicular fluid without additional washing. A brief washing step in a culture medium (Multipurpose Handling Medium (MHM, 90163, Irvine Scientific) supplemented with SSS (99193, Irvine Scientific), MHM Complete (90166, Fujifilm, Irvine Scientific), Sydney IVF Fertilization medium (Cook Medical, K-SIFM-20), or Continuous Single Culture Complete Medium (CSCM-C, 90165, Fujifilm, Irvine Scientific) was considered necessary if the follicular fluid contained blood. Mural GCs were aspirated in a 2.5 μL volume with a sterile tip (ep Dualfilter T.I.P.S. 10 μL S, Eppendorf, Wesseling-Berzdorf, Germany), transferred to 1.5 mL tubes (Sarstedt, Nümbrecht, Germany) pre-filled with 12–13 μL of RNAlater stabilization solution (Ambion, AM7020, Life Technologies, Carlsbad, CA, USA), and stored at 4 °C.

### 2.4. RNA Extraction

GCs in RNAlater were centrifuged at 5000× *g* for 5 min, and the supernatants were removed. Total RNA was isolated from these GCs using TRIzol (Life Technologies by Thermo Fisher, Carlsbad, CA, USA) according to the manufacturer’s instructions [38,39] with PEQGOLD PHA-SETRAP A 1.5 mL tubes (VWR International GmbH, Darmstadt, Germany) or MaXtract^TM^ High Density 1.5 mL tubes (Qiage Germantown, MD, USA), as described previously [17]. RNA was dissolved in RNase-free water, and concentration and purity were determined using a NanoDrop 2000c UV-spectrometer (NanoDrop Products, Wilmington, DE, USA). cDNA of the mRNA fraction was synthesized after oligo-dT priming with the SuperScript III First-Strand Synthesis System (Invitrogen by Life Technologies, Carlsbad, CA, USA) and M-MLV Reverse Transcriptase, RNase H Minus, Point Mutant (Promega, Madison, WI, USA).

### 2.5. Gene Expression Analysis

TaqMan predesigned gene expression assays for FMR1 (Hs00924544_m1), AKT1 (Hs00178289_m1), FOXO3 (Hs00818121_m1), FOXO1 (Hs00231106_m1), mTOR (Hs00234508_m1), S6K (Hs00177357_m1), TSC2 (Hs01020387_m1), and two housekeeping genes, HPRT and TBP (Hs99999909_m1; Hs00427620_m1, respectively), as well as the TaqMan universal PCR master mix, were purchased from Applied Biosystems (Life Technologies). Experiments were performed according to the manufacturer’s instructions. All samples were analyzed in triplicate with standard qPCR conditions using the Fast Forward 7500 Real-Time PCR System (Applied Biosystems, Life Technologies). Relative gene expression was analyzed using the ΔΔCt method [40]. The cDNA sample obtained from COV434 GCs [41] was used as an internal calibrator in each run.

### 2.6. Statistical Analysis

Data distribution was first determined using the Shapiro–Wilk test. For a simple comparison between ovarian reserves (NOR and POR), Student’s *t*-test or the Mann–Whitney test was used. Correlation was calculated using the Shearman–Rho correlation coefficient, as not all analyzed genes were normally distributed. Regression curves were tested for variable models for the AKT/mTOR signaling and AKT/FOXO signaling genes’ expression values with respect to *FMR1* expression values, patient age, and total rFSH or total HMG dose. The CGG subgroups and analysis of cohort demographic data were compared using a one-way analysis of variance or Kruskal–Wallis test as required. The adjusted Chi-square test was used for group comparisons (clinical ovarian response and IVF/intracytoplasmic sperm injection treatment). Results are presented as means ± standard deviations or median and interquartile range (25th–75th percentile; 1st–3rd quartile). For *n* < 3, data are presented as median and minimum and maximum values (minimum–maximum). Statistical analyses were performed using the Statistical Package for the Social Sciences V. 22.0 and 27 (IBM Corporation, Armonk, NY, USA), and statistical significance was set at *p* < 0.05.

### 2.7. CGG Repeat Analysis

Briefly, DNA samples were obtained from 10 mL of blood samples with EDTA, as described previously [17]. CGG repeat length in the 5′-UTR of *FMR1* exon 1 was analyzed by polymerase chain reaction (PCR) analysis and subsequent analysis of this region was carried out with the ALFexpress DNA sequencer (Amersham 1050, Pharmacia Biotech, Freiburg, Germany) or ABI 3100/3130xl sequencer (Life Technologies/Applied Biosystems, Foster City, CA, USA). PCR mixture (total volume, 30 mL) contained 0.25 μM of each primer (for forward and reverse primer sequences see Fu et al., 1991), 0.2 mM of dATP, dCTP, and dTTP each, 50 μM dGTP, 150 μM deaza-dGTP, 0.12 U KAPA Hot Start Taq polymerase, 1X PCR buffer, 1.5 mM MgCl_2_, 1X Enhancer (Qiagen GmbH, Hilden, Germany), and 50 ng of genomic DNA. PCR conditions were as follows: 3 min at 94 °C for the first denaturation step; 35 cycles of amplification with a time-temperature profile of 15 s at 94 °C, 15 s at 66 °C, then 15 s at 72 °C; and additional incubation for 8 min at 72 °C in the last cycle. The forward primer was labeled with the fluorescent Cy5 or FAM dye (Eurofins Genomics, Ebersberg, Germany). For analysis using the ALFexpress sequencer, a 5 μL aliquot of PCR mix was mixed with 5 μL of 6× loading solution (5 mg/mL Blue Dextran (Carl Roth GmbH + Co. KG, Karlsruhe, Germany) in formamide (Merck KGaA, Darmstadt, Germany) and 1 μL of 250-bp internal marker. All samples, following denaturation at 95 °C for 5 min, were analyzed in 6% denaturing polyacrylamide gel with 7 M urea. A 70–397 nucleotide size marker labeled with Cy5 dye was used for the determination of CGG repeat numbers. Allele sizes and peak areas of fluorescent products were analyzed with the Fragment Manager software (Pharmacia Biotech, Uppsala, Sweden). To analyze the samples on the ABI 3100/3130xl sequencer, 1 μL of PCR product was mixed with 10.5 μL of Hi-Di-formamide and 0.5 μL of GeneScan ROX standard (Applied Biosystems, Foster City, CA, USA) and loaded. Data were analyzed with the GeneMapper software (Applied Biosystems, Foster City, CA, USA). When the presence of PM was suspected, a Southern blot was performed using a 32P-dCTP radioactively-labeled p2 probe containing *FMR1* exon 1 with CGG repeats, as described previously [42].

### 2.8. Non-PM Allele Length Classification

CGG repeat lengths were categorized according to previous studies [19,43]. Patients were classified according to the repeat lengths at both alleles (low: < 26 repeats; normal: 26–34 repeats; high: 35–55 repeats) into six different genotypes: high/high, high/low, normal/high, normal/normal, normal/low, and low/low. The genotype high/high was not present in any patient, nor was PM with alleles over 54 repeats.

## 3. Results

### 3.1. General Study Population

A total of 84 POR and 225 NOR samples were included. The demographics are summarized in Table 1. The two groups differed significantly in AFC, AMH, age, total number of oocytes received, and number of mature MII oocytes, but not in BMI, nor in estradiol, FSH, or LH levels.

### 3.2. Gene Expression Analyses, Correlation and Regression Curve Models

In all patients, mRNA expression levels of *AKT1, TSC2, mTOR*, *S6K* and *FOXO3* had a positive and statistically significant correlation with *FMR1* mRNA expression level (*p* < 0.01 for all parameters) (Table 2).

When analyzed based on ovarian response, correlations within the groups of NOR and POR remained significant (Table 3 and Table 4), demonstrating significant correlation coefficients in both groups as well, particularly for *AKT1, mTOR*, *TSC2,* and *S6K.*

However, for *FOXO1,* no correlation was detected in their expression levels, nor with *FMR1* expression levels. This was consistent for all patients, as was confirmed after subgroup analysis considering the ovarian response (Table 2, Table 3 and Table 4). Moreover, for *FOXO3* in POR, significance was lost.

In addition, generalized regression curves for variable regression models were produced and are included as supplemental figures (Supplemental Figure S1–S6). They demonstrate, for *AKT, mTOR, TSC2, S6K,* and *FOXO3* expression values, highly significant regression curves (ANOVA *p* < 0.000) with *FMR1* expression, with the linear model being the best fitting one compared to other models. *FOXO1* showed no significant regression for any of the analyzed models with *FMR1*. Moreover, when analyzing the expression of the genes with respect to age and total rFSH or HMG dose, none of the regression models led to significant results (ANOVA *p* values n.s. for all).

**Figure 1:**
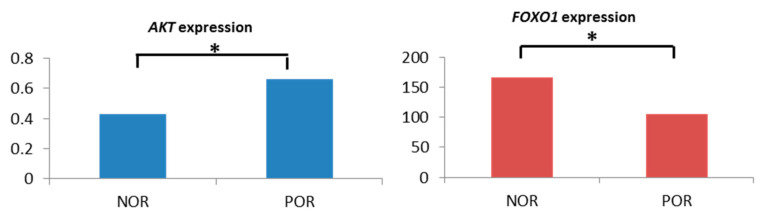
Significant expression alteration of *AKT1* (left figure) and *FOXO1* (right figure) in patients according to different ovarian response (NOR vs. POR). * significant difference. *X*-axis: ovarian response group (NOR: normal responder; POR: poor responder); *Y*-axis: median of relative gene expression.

*AKT1* demonstrated a significantly higher expression in the POR group (*p* = 0.040), whereas its downstream element *FOXO1* demonstrated a significantly lower expression in POR (*p* = 0.002), compared with the NOR group (Table 5, Figure 1).

For *TSC2, mTOR*, *S6K*, and *FOXO3* expression levels, no significant differences between NOR and POR groups were detected (Table 4).

### 3.3. CGG Repeat Analysis and Gene Expression Values

Distribution of different *FMR1* genotypes between NOR and POR groups was comparable (Table 6). No PM was detected.

To exclude the impact of CGG repeat length on our results, relative gene expression levels of *AKT1, TSC2, mTOR*, *S6K, FOXO1*, and *FOXO3* were subdivided into different *FMR1* genotypes (high/high, high/low, normal/high, normal/normal, normal/low, and low/low) and ovarian response groups (NOR and POR). Expression of the key genes of the AKT/mTOR signaling pathway, i.e., AKT and FOXO1/3 in the different *FMR1* genotype subgroups was not significantly different in our analysis, in none of the response groups (Supplementary Table S1).

## 4. Discussion

Our study data indicate a functional interplay between *FMR1* expression rate and the expression of key genes of the AKT/mTOR signaling pathway in native human GCs, with impact on ovarian response. We confirmed comparable in vitro data based on the COV434 cell line [32] using ex vivo GC samples. Clinically, NOR and POR samples are distinguished by differences in AFC, AMH, number of total oocytes received, and number of mature MII oocytes. Both patient groups displayed similar BMI and basal estradiol, FSH, and LH levels. Variable *FMR1* genotypes also did not impact the expression levels of the analyzed AKT/mTOR or AKT/FOXO signaling pathway genes. 

During the highly orchestrated molecular processes of human folliculogenesis and oocyte maturation, GCs continuously interact with oocytes and directly control the fate of follicles. They are, therefore, of utmost importance in follicular maintenance and growth, as well as for follicular atresia controlled by apoptotic processes [44]. 

The AKT/mTOR signaling pathway plays a role in diverse cellular functions and initiates the transcription–and regulates the translation–of multiple genes in many cell types. It also plays roles in autophagy and the biosynthesis of lysosomes and lipids [45]. In this pathway, AKT acts as a prominent protein phosphorylation kinase. In human ovaries, it is expressed in oocytes and GCs. Accordingly, in the mouse AKT isoform, AKT1 is involved in fertility control [27,28]. AKT phosphorylates TSC2, which then forms a dimer with TSC1 to induce mTORC formation. Subsequently, mTORC phosphorylates S6K, a major kinase in a further cell-specific translational control mechanism. In human neuronal cells, elevated S6K expression induces FMRP phosphorylation [36]. The AKT/mTOR signaling pathway also regulates GC differentiation and proliferation and is thus functional for ovarian activation [46].

In this study, we demonstrated that *FMR1* expression in native human GCs in women with different ovarian responses is significantly and positively correlated with the expression levels of *AKT1, TSC2, mTOR,* and *S6K.* Correlations between *FMR1* expression and the AKT-FOXO1/3 signaling pathway genes were not apparent, especially in the POR patient group. Results were consistent after regression curve analysis for all genes. These results thus support a specific functional linkage between *FMR1*/FMRP expression and AKT/mTOR signaling in human GCs, supporting the results of our previous study on the COV434 granulosa cell line [32]. Putative confounders, i.e., age and total rFSH or HMG dose, were excluded, as no significant regression curve was apparent for any of the analyzed genes´ values with respect to those factors.

Elevated *FMR1* expression levels cause reduced FMRP levels by a negative feedback loop mechanism [14]. Because FMRP binds *Tsc2* and *mTor* within the RISC [33], decreased FMRP levels will suppress the binding of GCs in the RISC. This results in the increased expression of unbound AKT/mTOR gene products, activating GC differentiation and proliferation, and further follicular development. This hypothesis was further supported by the results of the POR patient group, where *S6K* correlation appeared to be more pronounced. This may reflect a compensational reaction to the lower ovarian reserve, although proof of such a causality needs to be evaluated in future studies to discriminate between an *FMR1* expression only or an *FMR1* and ovarian response-dependent mechanism. Nevertheless, our results are also in line with those seen in elevated mTOR and S6K levels in *Fmr*-KO mice, which led to the premature recruitment of the oocyte pool [47], and are comparable with the results for lower AMH level (POR), where increased follicular activation as a compensatory mechanism is suggested [48].

AKT expression is the bridge between mTOR and FOXO signaling. In the latter, it induces FOXO phosphorylation, leading to their exclusion from the nucleus into the cytoplasm and, thereby, to their inactivation [49]. Decreased AKT expression thus results in dephosphorylation of FOXO1/3, which then shuttles back into the nucleus. There, it induces a proapoptotic pathway with DNA fragmentation, granulosa cell death, and, finally, follicular atresia, as well as oocyte silencing [44]. Consequently, women with POI were offered AKT activators, which lead to follicular activation [50].

In this study, *FOXO1/3* expression was analyzed in fresh GCs of patients with different ovarian responses. Of these, *FOXO1* is highly expressed in human GCs [51]. Although we found no correlative effect of *FMR1* on the expression levels of these *FOXOs*, at least in the poor responding group, an inverse gene expression activation of *AKT1* and *FOXO1* depending on ovarian response has been identified; higher *AKT1* and lower *FOXO1* expression were apparent in POR, and the reverse was true in NOR. Comparable results were shown in recent animal studies [52,53]. These studies suggest an opposing *AKT1*-triggered and *FMR1*-independent regulating mechanism that controls the ovarian reserve. Downregulated *FOXO1* in POR may thus prevent increased proapoptotic events to maintain the already limited ovarian reserve.

### Limitations

Our results are limited by the sample size. The number of GCs obtained per puncture is limited as well. In addition, we used GCs only from preovulatory follicles, which may differ from GCs of other, earlier stages of development. Patient ages were slightly different in both groups, with POR patients older than NOR patients by an average of three years. However, both patient groups had a mean age below 40 years. Nevertheless, an age-related effect cannot be excluded. As granulosa cell numbers per patient and per puncture are limited, expression analysis was not possible for all genes in every patient, most notably for *TSC2* and *FOXO1.*

Therefore, further studies including more clinically defined NOR and POR patients, subdivided into different age groups, are needed to support the suggested functional *FMR1*/AKT/mTOR linkage and its impact on female fertility.

## 5. Conclusions

We confirmed a functional linkage between *FMR1* expression and the AKT/mTOR signaling pathway genes in native human GCs, which was first revealed in the COV434 granulosa cell line. This linkage is only found with the key genes of the AKT/mTOR signaling pathway. AKT/FOXO1/3 signaling was not associated with *FMR1* expression in poor responding patients. This *FMR1*/AKT/mTOR linkage is, therefore, probably specific to follicular maturation control and indicates a putative, ovarian reserve-dependent balancing mechanism in the female germline.

## Figures and Tables

**Table 1:** Demographics of study cohort.

Demographic	NOR		POR		*p* Value
	*n*	Median (P25-P75)	*n*	Median (P25-P75)	
Age	225	34.7 ± 4.1 ^a^	84	37.7 ± 4.6	<0.001 ^b^
BMI	223	23.1 (20.1–26.8)	80	22.48 (20.5–25.0)	0.182
AFC	108	13 (9–20)	37	6 (4–7.5)	<0.001
FSH (U/L)	209	7.4 (6.10–8.9)	73	8.0 (6.1–10.4)	0.076
LH (U/L)	214	5.4 (3.8–7.1)	77	5.2 (3.6–6.3)	0.133
Estradiol (pg/mL)	208	43 (34.8–54.0)	74	51.3 (35.6–78.5)	0.018
AMH (ng/mL)	216	2.5 (1.5–4.0)	82	0.8 (0.5–1.1))	<0.001
Total oocytes	225	9 (6–13)	82	4 (2–6)	<0.001
MII oocytes	157	7 (5–10.5)	51	3 (2–6)	<0.001

BMI: body mass index, AFC: antral follicle count, FSH: follicle-stimulating hormone, LH: luteinizing hormone, AMH: anti-Müllerian hormone, MII oocytes: mature oocytes. ^a^ Mean ± standard deviation. ^b^ Student’s *t*-test. All other values represent median values with first and third quartiles parenthesized. *p* values represent significance levels between normal responders (NOR) and poor responders (POR).

**Table 2:** Correlations of *FMR1* gene expression and *AKT1* (AKT serine/threonine kinase), *FOXO3* (forkhead box O3), *FOXO1* (forkhead box O1), *mTOR* (mechanistic Target of Rapamycin), *TSC2* (tuberous sclerosis protein 2), and *S6K* (ribosomal protein S6 kinase) gene expression in all patients. The relative gene expression in granulosa cells of patients was normalized by two housekeeping genes and a granulosa cell calibrator using the ΔΔCt method. Correlation was calculated using the Spearman–Rho (r) correlation coefficient.

All Patients	Spearman-Rho Correlation Co-Efficient *FMR1*	*p* Value
*AKT*	0.666	<0.01
*mTOR*	0.722	<0.01
*TSC2*	0.556	<0.01
*S6K*	0.654	<0.01
*FOXO3*	0.284	<0.01
*FOXO1*	−0.034	0.689

**Table 3:** Correlations of *FMR1* gene expression with *AKT1*, *FOXO3*, *FOXO1*, *mTOR*, *TSC2*, and *S6K* expressions according to ovarian response group (NOR). The relative gene expression in granulosa cells of patients was normalized by two housekeeping genes and a granulosa cell calibrator using the ΔΔCt method. Correlation was calculated using the Spearman–Rho correlation coefficient.

NOR Patients	Spearman-Rho Correlation Co-Efficient *FMR1*	*p* Value
*AKT*	0.672	<0.01
*mTOR*	0.731	<0.01
*TSC2*	0.584	<0.01
*S6K*	0.647	<0.01
*FOXO3*	0.263	<0.01
*FOXO1*	−0.003	0.972

**Table 4:** Correlations of *FMR1* gene expression with *AKT1*, *FOXO3*, *FOXO1*, *mTOR*, *TSC2*, and *S6K* expressions according to ovarian response group (POR). The relative gene expression in granulosa cells of patients was normalized by two housekeeping genes and a granulosa cell calibrator using the ΔΔCt method. Correlation was calculated using the Spearman–Rho correlation coefficient.

POR Patients	Spearman-Rho Correlation Co-Efficient *FMR1*	*p* Value
*AKT*	0.667	<0.01
*mTOR*	0.712	<0.01
*TSC2*	0.372	0.047
*S6K*	0.690	<0.01
*FOXO3*	0.337	0.05
*FOXO1*	−0.133	0.475

**Table 5:** *AKT1*, *FOXO3*, *FOXO1*, *mTOR*, *S6K*, and *TSC2* expressions according to ovarian response group. For statistical details, see Materials and Methods.

Demographic	NOR		POR		*p* Value
Gene	*n*	Median (P25-P75)	*n*	Median (P25-P75)	
*AKT1*	225	0.47 (0.22–1.00)	83	0.66 (0.37–1.25)	0.040
*TSC2*	105	0.183 (0.09–0.298)	29	0.233 (0.145–0.329)	0.392
*mTOR*	225	0.05 (0.02–0.13)	84	0.08 (0.03–0.14)	0.296
*S6K*	191	0.17 (0.12–0.24)	67	0.19 (0.13–0.26)	0.342
*FOXO3*	193	0.59 (0.36–0.98)	68	0.51 (0.29–0.82)	0.064
*FOXO1*	105	166 (117–243)	29	105 (95–146)	0.002

**Table 6:** *FMR1* genotype distribution of patients with different response patterns.

Frequencies	NOR—*n* (%)	POR—*n* (%)
high/high	0	0
high/low	4 (1.8)	0
high/normal	18 (8.3)	12 (15.0)
normal/normal	128 (59.0)	46 (57.5)
normal/low	59 (27.2)	20 (25.0)
low/low	8 (3.7)	2 (2.5)
	217 (100)	80 (100)

## Data Availability

Data are available on reasonable request from the corresponding author.

## Supplementary Materials

The following are available online at https://www.mdpi.com/article/10.3390/jcm10173892/s1, Figure S1: Regression Curves of *AKT1/FMR1* expression values, Figure S2: Regression Curves of *mTOR**/FMR1* expression values, Figure S3: Regression Curves of *TSC2/FMR1* expression values, Figure S4: Regression Curves of *S6K/FMR1* expression values, Figure S5: Regression Curves of *FOXO3/FMR1* expression values, Figure S6: Regression Curves of *FOXO11/FMR1* expression values, Table S1: Expression of the key genes of the AKT/mTOR signaling in different *FMR1* genotype subgroups.
