# Supplementary material for: FMR1 and AKT/mTOR Signaling in Human Granulosa Cells: Functional Interaction and Impact on Ovarian Response
Source: J Clin Med. 2021 Aug 30;10(17):3892. doi: 10.3390/jcm10173892 (PMC8432207; doi:10.3390/jcm10173892)
Supplement: Supplementary file 1 [file jcm-10-03892-s001.zip › jcm-1349189-supplementary.pdf]

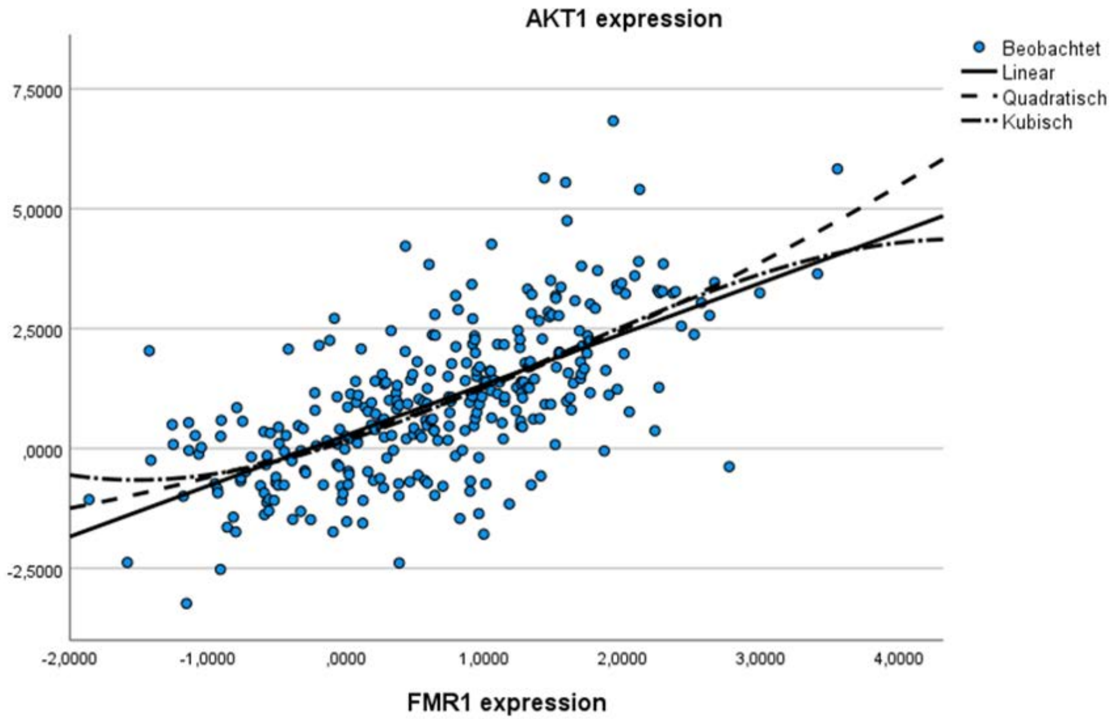

Figure S1. Regression Curves of *AKT1*/*FMR1* expression values.

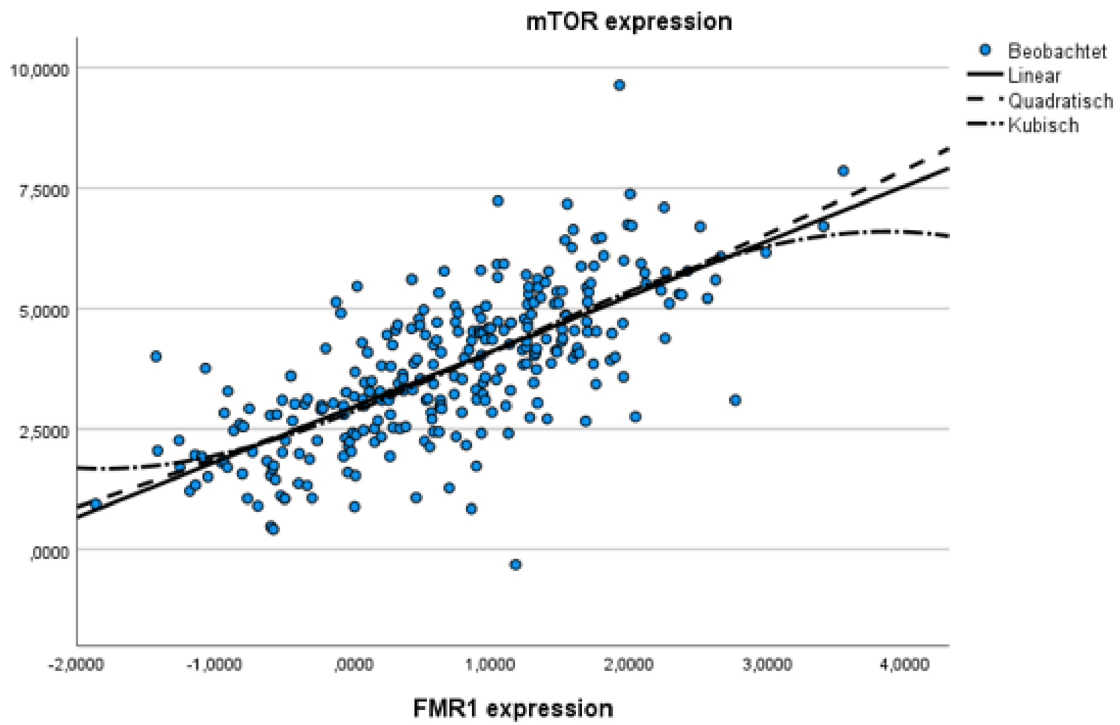

Figure S2. Regression Curves of *mTOR*/*FMR1* expression values.

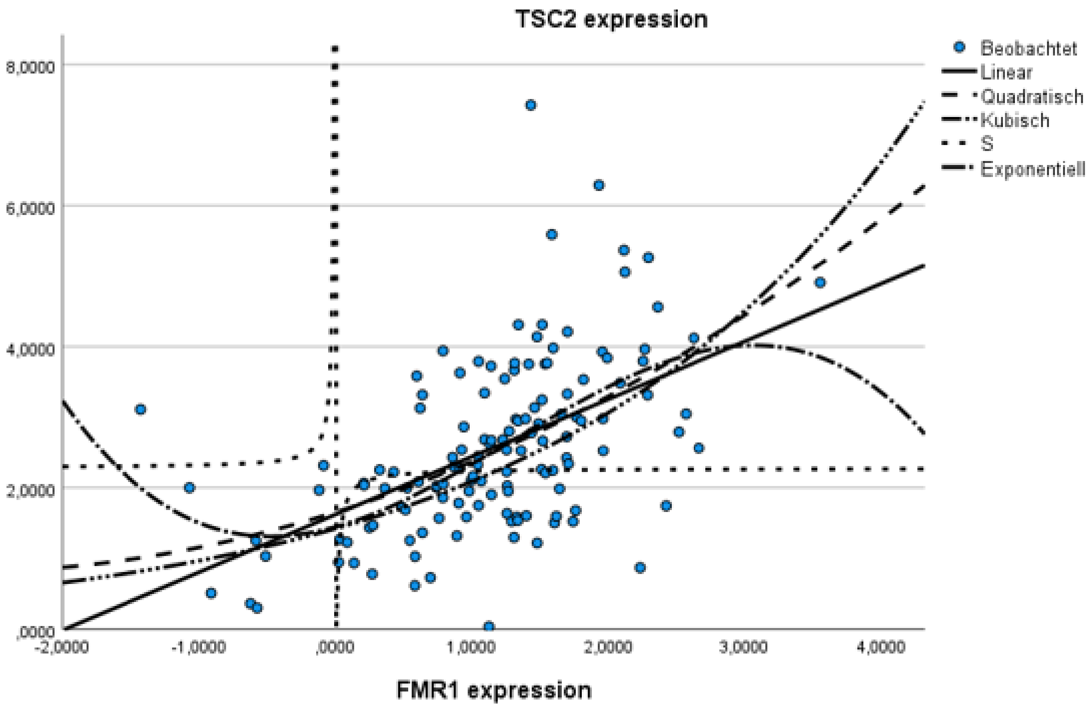

Figure S3. Regression Curves of *TSC2//FMR1* expression values.

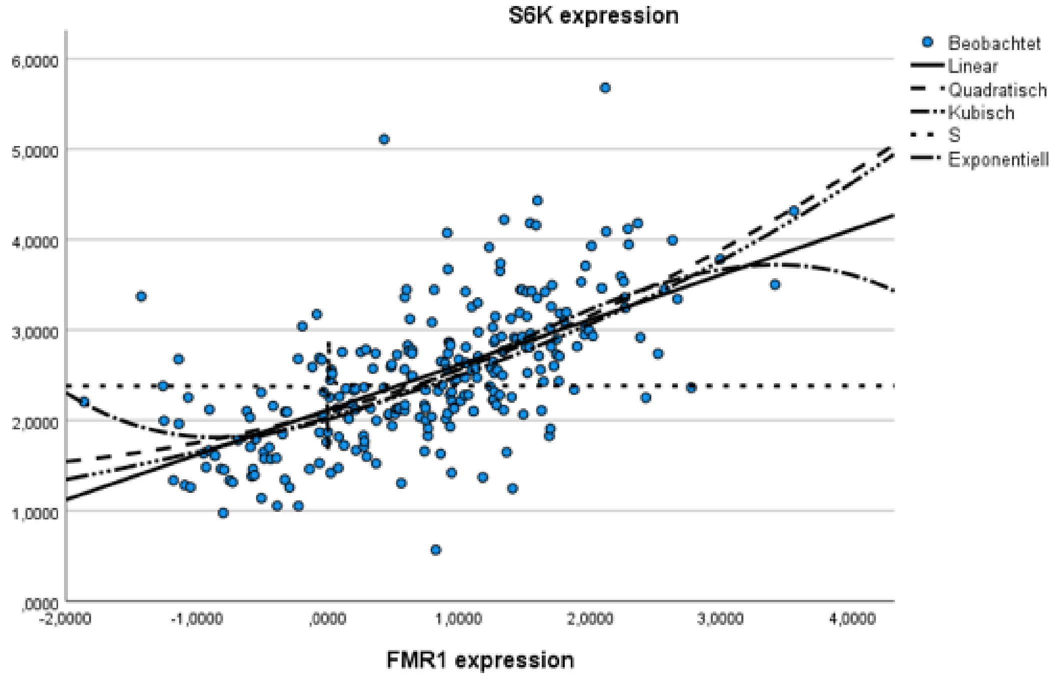

Figure S4. Regression Curves of *S6K*/*FMR1* expression values.

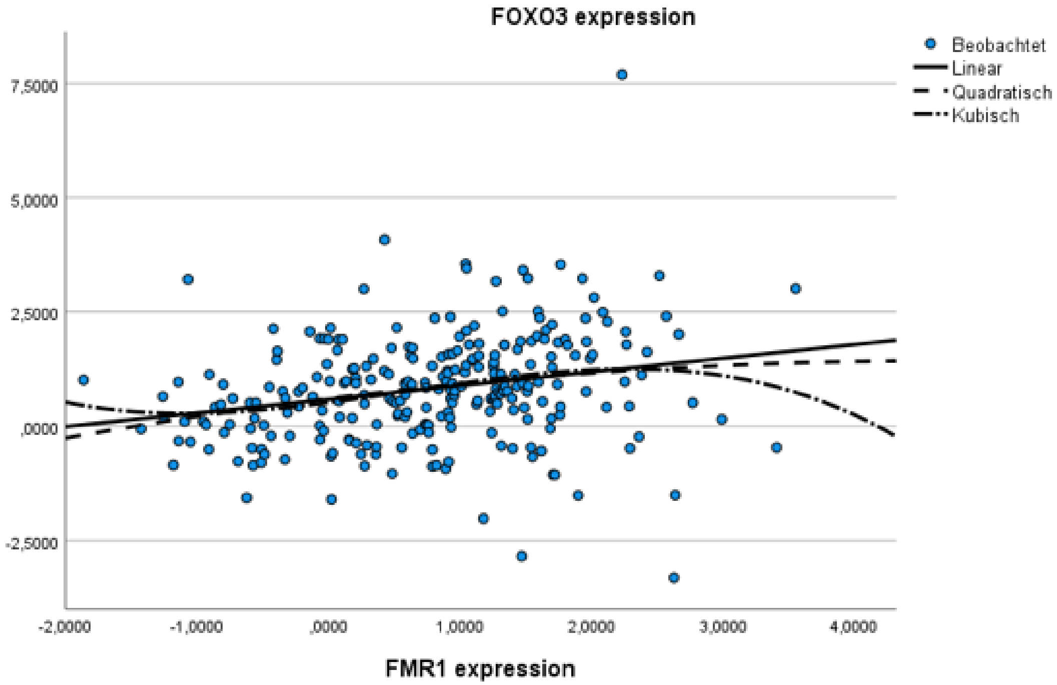

Figure S5. Regression Curves of *FOXO3*/*FMR1* expression values.

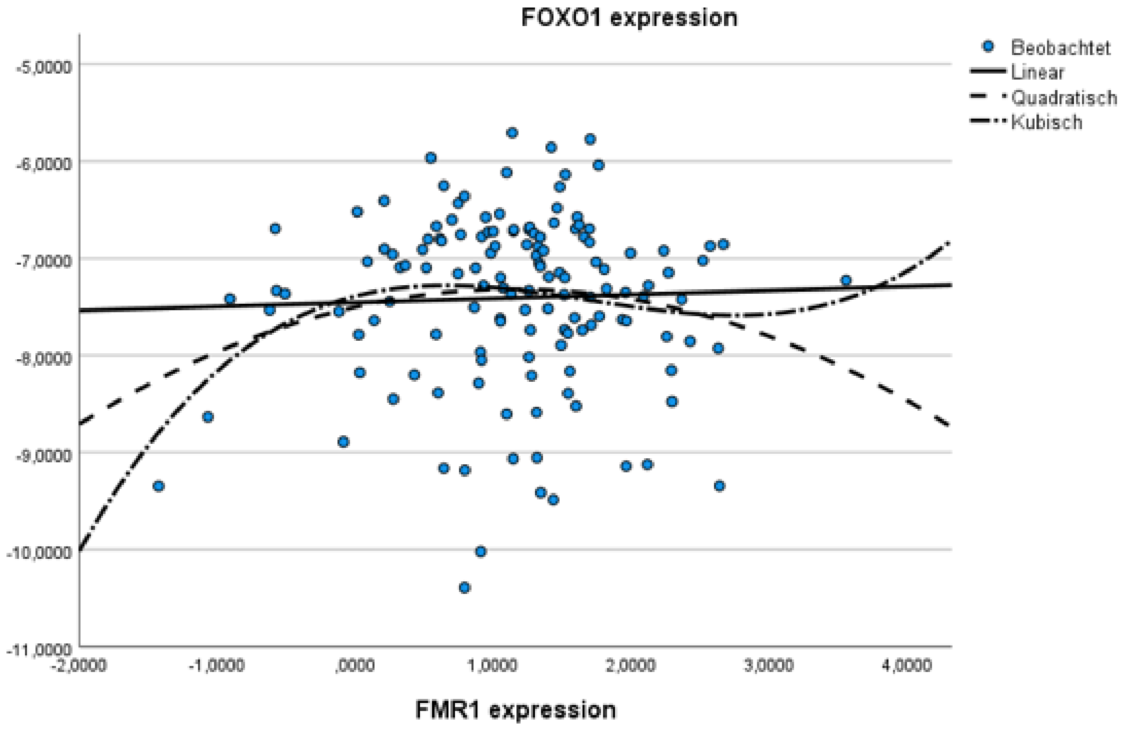

Figure S6. Regression Curves of *FOXO1*/*FMR1* expression values.

Table S1. Expression of the key genes of the AKT/mTOR signaling in different *FMR1* genotype subgroups

Genotype	high/low		high/normal		normal/normal		normal/low		low/low		*p* value
Gene expression	*n*	(P25-P75)	*n*	(P25-P75)	*n*	(P25-P75)	*n*	(P25-P75)	*n*	(P25-P75)	
AKT1											
All patients	4	1.0 (0.3 - 2.3)	30	0.6 (0.3 - 0.8)	174	0.5 (0.2 - 1.0)	79	0.7 (0.3 - 1.5)	10	0.3 (0.3 - 0.4)	0.068
NOR	4	1.0 (0,3 - 2.3)	18	0.6 (0.2 - 1.0)	128	0.4 (0.2 - 0.9)	59	0.5 (0.3 - 1.3)	8	0.2 (0.1 - 0.5)	0.274
POR	0	-	12	0.6 (0.4 - 0.8)	46	0.5 (0.3 - 1.0)	20	1.0 (0.6 - 1.6)	2	0.3 (0.2 - 0.4)	0.91
FOXO3											
All patients	3	1.4 (1.2 - 2.2)	25	0.6 (0.4 - 0.9)	153	0.5 (0.3 - 0.8)	59	0.5 (0.3 - 0.9)	10	0.3 (0.3 - 0.5)	0.064
NOR	3	1.4 (1.2 - 2.2)	15	0.7 (0.4 - 1.0)	115	0.5 (0.3 - 0.8)	45	0.5 (0.4 - 1.0)	8	0.4 (0.3 - 0.6)	0.137
POR	0	-	10	0.5 (0.4 - 0.7))	38	0.4 (0.3 - 0.7)	14	0.5 (0.3 - 0.8)	2	0.2 (0.2 - 0.2)	0.294
FOXO1											
All patients	3	108 (104 - 146)	12	142 (103 - 230)	81	155 (111 - 213)	27	125 (114 - 187)	4	150 (63 - 245)	0.773
NOR	3	108 (104 - 146)	8	152 (119 - 230)	62	164 (121 - 237)	22	133 (116 - 286)	4	150 (63 - 245)	0.602
POR	0	-	4	125 (103 - 265)	19	106 (97 - 152)	5	84 (76 - 119)	0	-	0.347
mTOR											

All patients	3	0.05 (0.04 - 0.16)	29	0.05 (0.03 - 0.13)	163	0.06 (0.03 - 0.14)	72	0.10 (0.04 - 0.18))	10	0.04 (0.01 - 0.12)	0.195
NOR	3	0.05 (0.04 - 0.16)	17	0.05 (0.02 - 0.11)	121	0.06 (0.02 - 0.14)	55	0.09 (0.04 - 0.16)	8	0.03 (0.01 - 0.11)	0.293
POR	0	-	12	0.06 (0.03 - 0.15))	42	0.09 (0.04 - 0.14)	17	0.12 (0.07 - 0.26)	2	0.08 (0.04 - 0.12	0.366
S6K											
All patients	3	0.23 (0.20 - 0.25)	25	0.21 (0.12 - 0,24)	152	0.18 (0.13 - 0.25)	58	0.18 (0.14 - 0.23)	10	0.19 (0.13 - 0.22)	0.904
NOR	3	0.23 (0.20 - 0.25)	15	0.21 (0.20 - 0.25)	114	0.18 (0.12 - 0.25)	44	0.17 (0.14 - 0.23)	8	0.19 (0.14 - 0.22)	0.890
POR	0	-	10	0.21 (0.21 - 0.22)	38	0.18 (0.15 - 0.27)	14	0.21 (0.14 - 0.34)	2	0.16 (0.09 - 0.22)	0.758
TSC2											
All patients	3	0.33 (0.33 - 0.55)	12		81	0.17 (0.09 - 0.25)	27	0.23 (0.12 - 0.35)	4	0.18 (0.11 - 0.25)	0.188
NOR	3	0.33 (0.33 - 0.55)	8	0.18 (0.06 - 0.27)	62	0.17 (0.09 - 0.25)	22	0.20 (0.11 - 0.34)	4	0.18 (0.11 - 0.25)	0.239
POR	0	-	4	0.29 (0.17 - 0.40)	19	0.18 (0.12 - 0.29)	5	0.24 (0.23 - 0.38)	0	-	0.345
